# Adaptive Real-Time Routing Protocol for (*m*,*k*)-Firm in Industrial Wireless Multimedia Sensor Networks †

**DOI:** 10.3390/s20061633

**Published:** 2020-03-14

**Authors:** Beom-Su Kim, Sangdae Kim, Kyong Hoon Kim, Tae-Eung Sung, Babar Shah, Ki-Il Kim

**Affiliations:** 1 Department of Computer Science and Engineering, Chungnam National University, Daejeon 34134, Korea; bumsou10@cnu.ac.kr (B.-S.K.); sdkim.cse@gmail.com (S.K.); 2 School of Computer Science and Engineering, Kyungpook National University, Daegu 41566, Korea; 3 Department of Computer and Telecommunications Engineering, Yonsei University, Wonju 26493, Korea; tesung@yonsei.ac.kr; 4 College of Technological Innovation, Zayed University, P.O. Box 144534, Abu Dhabi, UAE; Babar.Shah@zu.ac.ae

**Keywords:** (*m*,*k*)-firm model, adaptive real-time routing, traffic management, industrial wireless multimedia sensor network

## Abstract

Many applications are able to obtain enriched information by employing a wireless multimedia sensor network (WMSN) in industrial environments, which consists of nodes that are capable of processing multimedia data. However, as many aspects of WMSNs still need to be refined, this remains a potential research area. An efficient application needs the ability to capture and store the latest information about an object or event, which requires real-time multimedia data to be delivered to the sink timely. Motivated to achieve this goal, we developed a new adaptive QoS routing protocol based on the (*m*,*k*)-firm model. The proposed model processes captured information by employing a multimedia stream in the (*m*,*k*)-firm format. In addition, the model includes a new adaptive real-time protocol and traffic handling scheme to transmit event information by selecting the next hop according to the flow status as well as the requirement of the (*m*,*k*)-firm model. Different from the previous approach, two level adjustment in routing protocol and traffic management are able to increase the number of successful packets within the deadline as well as path setup schemes along the previous route is able to reduce the packet loss until a new path is established. Our simulation results demonstrate that the proposed schemes are able to improve the stream dynamic success ratio and network lifetime compared to previous work by meeting the requirement of the (*m*,*k*)-firm model regardless of the amount of traffic.

## 1. Introduction

Even though many typical Wireless Sensor Networks (WSNs) have been employed to detect the events and report interested values around itself, they are typically based on scalar values and therefore have limited abilities to obtain various types of information. This implies that limited information, such as the geographic position of the target, can be recorded. As an alternative, wireless multimedia sensor networks (WMSN) [1] can capture multimedia information about the object or event by using a camera and microphone. However, the more complicated nature of multimedia data (compared to scalar values) has prompted considerable research in an attempt to effectively capture the information as well as to deliver this information in real time. One of the good potential examples of WMSN is industrial sensor networks [2] to monitor and control systems. In industrial sensor networks, it is essential to employ QoS routing protocol in WMSN to accomplish the mission.

To address the problems associated with real-time delivery in WMSNs, several quality of service (QoS) routing protocols [3,4,5] have been proposed and discussed in the literature. However, most existing schemes still need to be improved by using a specific traffic model and applications. Based on general application model, many of them lack of applicability and lead to failure of QoS guarantee. To defeat this problem, the (*m*,*k*)-firm traffic model was recently presented for WMSNs. As a firm real-time model called (*m*,*k*)-firm is proposed to measure real-time application performance. The concept of (*m*,*k*)-firm is defined that a real-time message stream is considered to have an (*m*,*k*)-firm guarantee requirement that at least *m* out any *k* consecutive messages from the stream must meet their deadlines to ensure adequate QoS. However, the (*m*,*k*)-firm model has not been studied with respect to finding specific applications in which this model could be utilized. Thus, they have the same problem as general QoS routing protocol.

These issues motivated us to develop the adaptive real-time routing protocol we present in this paper. The model is based on the (*m*,*k*)-firm model [6,7], which serves the purpose of adding real-time capabilities. Unlike previous work, which focused on real-time delivery only, our previous approach is to introduce a multimedia application modeled by a (*m*,*k*)-firm stream. This new stream is maintained to ensure quality by using a new QoS routing protocol for the (*m*,*k*)-firm stream to reduce the overhead for path establishment and maintenance. To achieve this goal, based on the current status of application of the (*m*,*k*)-firm stream, a new QoS routing protocol was designed to select the path adaptively. When this routing protocol is unable to meet the requirement, an adaptive traffic-handling algorithm is employed. The operations of these two functions, which are dependent on the current status of the (*m*,*k*)-firm streams, ensure that high adaptability is achieved. Finally, extensive simulation results show that the proposed scheme meets the requirements of the (*m*,*k*)-firm stream for application and also provides robustness in terms of network traffic.

The main contributions of this paper are summarized below.

- To recognize the shortcomings or limitations of QoS routing protocol in WMSN, we provide a comprehensive survey that investigates state-of-the-art research work and further research directions for applying application specific requirement to the QoS routing protocol.
- To overcome the problem of general routing protocol considering deadline, we take (*m*,*k*)-firm based routing protocol while considering application requirements which is modeled by (*m*,*k*)-firm stream together.
- Unlike most of existing approaches which are based on (*m*,*k*)-firm stream, we propose a new adaptive path selection and traffic shaping algorithm.
- The proposed scheme is compared to existing (*m*,*k*)-firm based QoS routing protocols. Through various simulations, we prove that the proposed approach can reduce the dynamic failure ratio without regard to the background traffic as well as extend network lifetime.

The remainder of this paper is organized as follows. In Section 2, we discuss existing research on WMSNs and real-time routing protocols. In Section 3, we explain the real-time model, new QoS routing protocol, and traffic handling scheme. The results of the performance evaluation are provided in Section 4. Finally, the proposed scheme is summarized and further work is mentioned in Section 5.

## 2. Related Work

In this section, we present the related work for real-time routing protocols in WMSN. In addition to WMSN introduction, we present the outstanding potential applications including object tracking, in WMSN. Furthermore, previous real-time routing protocols are analyzed.

### 2.1. Wireless Multimedia Sensor Networks and Its Applications

In WMSN, each sensor node is equipped with visual and audio information collection modules such as microphones and video cameras. These nodes can retrieve multimedia content from the networks at variable rates and deliver the captured information through multi-hop communication to the sink. As compared to typical WSN, there are many research challenges that must be addressed such as high packet loss rate, shared channel, limited bandwidth, high variable delays, and lack of fixed infrastructure in WMSN. To overcome the mentioned deficiency, lots of research have been conducted in the aspects of algorithms, protocols, and hardware for the development of WMSN. Moreover, WMSN is expected to be employed increasingly for the potential applications such as surveillance and monitoring applications.

Among them, object tracking, which has been widely used and deployed in many applications, consists of object detection, object classification, and object reporting schemes. Considering these functions, wireless sensor networks (WSNs) become the potential architecture on which to implement object tracking. This is because WSNs are capable of detection and delivery at the same time in a cost efficient way. In addition, there are many applications on the WMSN such as road traffic monitoring, health-care applications, and area monitoring, etc. Road traffic monitoring [8,9] is defined as monitoring the behavior of vehicles on the road. Road traffic forecasting is important to the safety of the driver, traffic management and also fuel saving. As a promising application for real-time application, power plant [10] and port docks [11] can be monitored through deploying wireless multimedia sensor networks as well as real-time streaming to deal with emergency. Through the health-care applications [12,13], doctors and caregivers could monitor the patients’ condition and activities, remotely. These two tracking and monitoring application can be applied into industrial environment by preventing unexpected events and accidents in an efficient way. As an example, Dobslaw et al. [14] proposed a new SchedEx-GA, integrated cross-layer framework, to admit a network configuration. Moreover, Sun et al. [15] proposed end-to-end data delivery reliability (E2E-DDR) to estimate and optimize the reliability in industrial sensor networks. They introduce a new mapping function with the packet reception ratio, background noise, and received signal strength by modeling background noise and path loss model. On the other hand, Zhang et al. [16] proposed an energy-efficient QoS routing algorithm along reliable path by classifying the industrial sensing data into three data types and setting their priority. This classification is used to establish forwarding node set and strategies to guarantee QoS in the aspects of reliability and real-time.

There are some research work to enhance the performance of WMSN. At first, Sarisaray-Bolukand and Akkaya [17] presented how to utilize background subtraction and compression techniques to reduce data. They analyzed the previous work in the aspects of computation and communication energy, time, and quality and provide quantitative experimental results for packet loss on the Android platform. In addition, Bavarva et al. [18] proposed how to improve system performance through multiple input multiple output along with compressive sensing. With their help, reduced energy consumption and acceptable image quality are achieved.

### 2.2. Real-Time Routing Protocols

The real-time transmission is essential for various applications such as multimedia, disaster response, and various studies have been proposed according to the demand. To achieve real-time transmission, the packets should be delivered to their destination within the required time of the applications. In this section, we describe previous work related to real-time routing in WSNs.

SPEED [19] has proposed a spatiotemporal approach for the real-time service. SPEED could satisfy the real-time requirement by maintaining a desired delivery speed across the networks. The desired delivery speed depends on not only the end-to-end distance but also the desired time. The intermediate node relays the packet through the node which satisfy the desired speed. As an extension of SPEED, SPEED-Realtime Routing (RR) [20] has been proposed to comprise between real-time and energy cost. To decide the next hop, three metrics, that is, residual energy of two-hop neighbor nodes, routing void and rate void rate are taken. Furthermore, adaptive control for data rate and cache queue length is employed to distinguish the congestion control. ORRP [21] presented an opportunistic real-time routing scheme. ORRP broadcasts the packet to the neighbor nodes which satisfy the real-time requirement. All neighbors received packet could obtain the priority according to a tolerable time period to be able to satisfy the requirement. As the ORRP could offer the transmission opportunity more neighbors, it could achieve reliable real-time packet transmission. Yang et al. [22] addressed that the remaining time gain could exploit to satisfy the real-time requirement. In the transmission process, the intermediate node accumulates the remaining time to gain through selecting nodes that have a higher speed than the desired speed. The accumulated time would be exploited to pass the area which no neighbors meet the real-time requirement. Unlike the previous work based on single path approach, Hassen et al. [23] presented a comprehensive multipath routing approach for QoS in WMSN. Their main strategy for survey was design issues in the aspects of multimedia data. Park et al. [24] presented a disjointed multipath routing scheme for real-time data transmission in WMSN through hybrid approach with Bluetooth and Zigbee to overcome the bandwidth limitation. Moreover, Deepaa and Sugunab [25] proposed Optimized QoS-based Clustering with Multipath Routing Protocol (OQoS-CMRP) to determine cluster heads while considering sink coverage area and energy. In addition, Kim et al. [26] presented the opportunistic multipath routing to improve the performance in WSN which can be applied to multimedia data delivery.

However, these approaches do not include real-time delivery of captured multimedia data and would need to be extended. In this regard, Kim and Sung [27] proposed cross layered approach for (*m*,*k*)-firm stream in WSN. In each layer, transmission power, priority of packet and QoS path is adjusted and found. Moreover, Li and Kim [28] presented (*m*,*k*)-firm based routing protocol which is based on a set of fault recovery mechanisms to overcome inherent resource constraint of sensor nodes and instability of wireless communication. A local status indicator to monitor and evaluate their local conditions is introduced on intermediate node. Kim et al. analyzed the challenges presented by real-time communication [29] as part of special issue [30]. Specifically, they introduced a firm real-time model together with hard and soft models. As representative examples, Kim and Sung [31] presented a distributed, measurement-based scheme for the (*m*,*k*)-firm stream. In addition, they extended this approach to address the scalability problem and lack of architecture for the (*m*,*k*)-firm stream by using flow aggregation based on a compositional hierarchical model, a velocity-based routing protocol, a hybrid medium access control protocol, and a congestion control scheme [32]. Azim et al. [33] presented a new real-time routing protocol for a (*m*,*k*)-firm model by introducing multiple attributes to evaluate the path. In addition to this, Park et al. [34] proposed a new tree-based broadcast protocol to meet the requirements of the (*m*,*k*)-firm stream in WSNs. They focused on energy efficiency by selecting as few as nodes on a tree as possible and a fast recovery scheme based on (*m*,*k*)-firm real-time conditions and local states monitoring. However, these existing schemes focus on the real-time routing based on networks environments without the considering the application requirements. This implies that a path is selected by depending on Distance Based Priority (DBP) value of (*m*,*k*)-firm stream without the quality of application.

The analysis we provide above indicates that most previous schemes would need to be extended to enable real-time delivery. Furthermore, a multimedia traffic model for streaming application would need to be introduced. Additionally, the severely constrained capabilities of sensor nodes in terms of their energy, memory, and processing power demand an adaptive approach to meet the requirements of varying network parameters.

## 3. Adaptive Real-Time Routing Protocol

In this section, we describe our real-time model for application and the adaptive QoS routing protocol to enable real-time processing.

### 3.1. Real-Time Model

**Figure 1:**
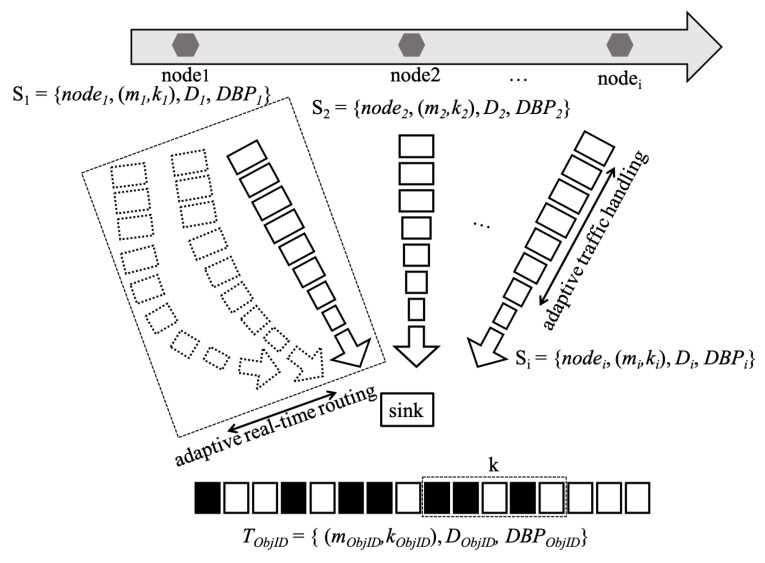
Overview of the proposed real-time model.

We aim to model the application of multimedia data traffic and a real-time routing protocol to deliver traffic timely. We assume the event happens continuously and is recognized by a node. We focus on traffic modeling and real-time delivery. The network architecture, including the nodes, is illustrated in Figure 1, which also shows that a sink node exists in the network. Traffic model and requirements are as follows.

A node is capable of processing consecutive images and transmitting them to the sink node with the identity unique ObjID. The application stream, $T_{ObjID}$, is modeled by the (*m*,*k*)-firm model, that is, $T_{ObjID}$ = {$m_{ObjID}$, $k_{ObjID}$, $D_{ObjID}$, $DBP_{ObjID}$} where $m_{ObjID}$ and $k_{ObjID}$ represent the requirements of a multimedia application. In addition, if the packet is transmitted within the deadline, $D_{ObjID}$, the corresponding packet is marked as Hit; otherwise, it is marked as Miss. Here, $DBP_{ObjID}$ is prioritized value based on the distances of the stream requirement to recognize the current (*m*,*k*)-firm status. For example, with a (3,5)-firm real-time stream requirement, the traces of the reception status of the last 5 packets can be represented as HHMMM where H and M represent the Hit and Miss of status of an individual packet, respectively. Then, the DBP value becomes −1 as the required number of Hit is 3 where the Hit status remains only 2 as explained in our previous paper [35].

In addition to application stream, a real-time session flow between node *i* and the sink, denoted by $S_{i}$, is defined as $S_{i}$ = {$node_{i}$, $m_{i}$, $k_{i}$, $D_{i}$, $DBP_{i}$} like $T_{ObjID}$. The different element between $T_{ObjID}$ and $S_{i}$ is node identifier, $node_{i}$. Even though two model consists with the same parameters, the value for them is different according to requirements. $T_{ObjID}$ represents the application requirement while $S_{i}$ does requirement for routing protocol.

Before an event end at node *i*, $S_{i}$ is maintained and packets are transmitted to the sink by filling $T_{ObjID}$ with them through multiple paths as illustrated in dotted rectangle in Figure 1. According to current status of $D_{ObjID}$ and $DBP_{i}$, one of multiple paths are selected to transmit the packets. Each session is used to partially build $T_{ObjID}$. Each session has the same ($m_{i}$, $k_{i}$) requirements in the $T_{ObjID}$. The same values for $D_{i}$ are assumed for $T_{ObjID}$ and $S_{i}$. As illustrated in Figure 1, $T_{ObjID}$ consists of each consecutive sessions, that is $T_{ObjID}$ = {$S_{1}$∪$S_{2}$∪ … ∪$S_{n}$}. Thus, $DBP_{ObjID}$ is affected by $DBP_{i}$ as well as ($m_{ObjID}$, $k_{ObjID}$) value.

To meet the requirements of the two (*m*,*k*)-firm models, the values are assigned to comply with the following conditions. These two conditions guarantee that the requirement of $S_{i}$ cannot exceed that of the $T_{ObjID}$. This is because, occasionally, the network cannot ensure the time requirement because of severe constraints.

(1) $$\frac{m_{i}}{k_{i}}>\frac{m_{ObjID}}{k_{ObjID}}$$

(2) $$k_{i}\geq k_{ObjID}$$

Upon detecting any event, a node *i* builds a multimedia stream by using consecutive packets with the (*m*,*k*)-firm model and delivers them to the sink. The sink checks whether a packet is lost or whether the packet has arrived after the deadline. These measurements are used to compute the values of $DBP_{i}$ and $DBP_{ObjID}$, which are then transmitted to the node *i*.

### 3.2. Adaptive QoS Routing Protocol

An adaptive approach to QoS routing is taken according to $DBP_{i}$ in $S_{i}$ as well as $DBP_{ObjID}$ in $T_{ObjID}$ to meet the requirements of the (*m*,*k*)-firm in terms of both $T_{ObjID}$ and $S_{i}$. The QoS routing algorithm is presented in Algorithm 1.

Algorithm 1 determines a suitable path for the next packets among multiple paths. To find the paths, the node sends a probing packet to identify the available paths and waits for the end-to-end delay from the sink. The probing packet is broadcasted in the same as the Route Request message (RREQ) and Route Response (RREP) in ad hoc on-demand multipath distance vector (AOMDV) [36]. The difference between the proposed and AOMDV comes from the deadline. If an intermediate node receives the packet with greater elapsed time than deadline, $D_{i}$, it does not broadcast the packet any more. In addition, the sink node replies only when the end-to-end delay is bounded within $D_{i}$. Multiple paths meeting the delay constraint are arranged in ascending order and are denoted by $P_{i}^{1\ldots n}$. Based on the delay, the shortest path is denoted by $P_{i}^{1}$. Each node has a different maximum number of available paths, *n*. Initially, $P_{i}^{n}$ is selected according to the longest-path-first strategy. After the initial stage, a path is selected by $DBP_{i}$ in $S_{i}$ as well as $DBP_{ObjID}$ in $T_{ObjID}$. The path selection is accomplished whenever a reply with the value of $DBP_{ObjID}$ is received.

**If the value of $DBP_{ObjID}$ is positive (lines 5–13):** First, if $DBP_{ObjID}$ is larger than 0, the application requirement is regarded to be met. So, it is important to examine the value of $DBP_{i}$. If $DBP_{i}$ is larger than 0, application and session requirement are met. Thus, this path is suitable and complies with the two requirements. Otherwise, it is necessary to consider a new path with shorter delay among multiple paths, denoted by $P_{i}^{n-1}$ as in line 9. However, the current path needs to be maintained if no other available path remains, when n←1. As a result, if application requirement is met, a path is re-selected according to $DBP_{i}$ in $S_{i}$.

**If the value of $DBP_{ObjID}$ is negative (lines 14–29):** When the value of $DBP_{ObjID}$ is negative at node *i*, more complicated approaches than previous case are taken. This implies that additional options are taken into consideration to choose the path. That is, a new path is selected in aggressive way according to distance to positive status. To determine the distance to reach a positive status, we compute the absolute value with two DBP values. The larger value is obtained, the shorter path is taken. Similar to the previous case in which $DBP_{i}$ was positive, an incremental approach is taken for path selection simply. On the other hand, if the value of $DBP_{i}$ is also negative, we compare the absolute value with the threshold. If this value is larger than the threshold, the shortest path, $P_{i}^{1}$, is selected because current path is not suitable at all. This is to turn negative DBP value to positive one by ensuring the shortest delivery time between node *i* and the sink. Otherwise, another path is selected by choosing the path number, n−t, rather than n−1 as in the previous case. By applying n−t rather than n−1, it is possible to turn negative DBP to positive one in earlier than incremental approach.

We assume that the a new event on next node, *j*, happen after event time on node *i* is over. When the new node *j* detects the events, Algorithm 1 is initiated on a new node. However, the path selection algorithm takes some time to execute because multiple paths should be established through Path_Search_Probing(i,sink). To reduce the waiting time for a new path, node *j* temporarily sends packets directly to the previous node *i* until the new path is established. Node *i* transmits the corresponding packets along the path $P_{i}^{1}$. Upon establishing a path to node *j*, packets are delivered along $P_{j}^{n}$.
**Algorithm 1** Adaptive QoS routing protocol for packet transmission from node *i* to the sink.1: *n*← Number of available real-time paths2: $P_{i}^{1\ldots n}$← Path between node *i* and the sink where its end-to-end delay is bounded within $D_{i}$
▹$P_{i}^{1}$
 has the shortest delay whereas $P_{i}^{n}$ has the longest delay, $P_{i}^{1}$≤$P_{i}^{2}$≤ … ≤$P_{i}^{n}$ 3: *n*← Path_Search_Probing(i,sink) ▹ Obtain number of possible multiple paths from node i and sink4: Transmit packets along $P_{i}^{n}$▹ Packets are transmitted along the longest end-to-end delay in $P_{i}^{1\ldots n}$ 5: **for** every $DBP_{ObjID}$**do**▹ After obtaining $DBP_{ObjID}$ value of $T_{ObjID}$6:  **if**
$DBP_{ObjID}$ ≥ 0 **then**▹ If application stream meets the requirement7:   **if**
$DBP_{i}$ < 0 **then**▹ If the session stream is not meeting the requirment8:    **if**
n−1 > 0 **then**9:     Transmit packets along $P_{i}^{n-1}$▹ Change the path along alternative with the next  longest delay if possible10:    **else**11:     Maintain $P_{i}^{n}$▹ Current path is maintain if no alternative is available12:    **end if**13:   **end if**14:  **else**▹ If application stream doesn’t meet the requirement, more complex path selection  algorithm is performed15:   *t*← |$DBP_{ObjID}$ + $DBP_{i}$| ▹ Compute how many distance from positive status with two  DBP values16:   **if**
$DBP_{i}$ ≥ 0 **then**▹ if current session meets the requirement17:    **if**
n−1 > 0 **then**▹ if an available path exists18:     Transmit packets along $P_{i}^{n-1}$▹ Change alternative path among multiple paths.  Adaptive approach in the aspects of session19:    **else**20:     Transmit packets along $P_{i}^{1}$▹ Change path with the shortest end-to-end delay in case  of two requirements missing21:    **end if**22:   **else**▹ if current session and application don’t meet the requirement at the same time. This is  the worst case23:    **if**
*t* < Threshold **then**▹ Distance is not bounded within the threshold24:     Transmit packets along $P_{i}^{1}$▹ Change the path with the shortest end-to-end delay25:    **else**26:      Transmit packets along $P_{i}^{n-t}$▹ Change the ${\left(n-t\right)}^{th}$ path according to distance to  meeting status27:    **end if**28:   **end if**29:  **end if**30: **end for**

### 3.3. Adaptive Traffic Handling

An adaptive routing algorithm ensures the selection of a path with shorter end-to-end delay than the previous path according to both $DBP_{i}$ in $S_{i}$ and $DBP_{ObjID}$ in $T_{ObjID}$. However, if no other path is available, that is, $P_{i}^{1}$ is set to the current path, the adaptive traffic handling algorithm executes. The algorithm aims to reduce end-to-end delay by either redefining the ($m_{i}$, $k_{i}$)-firm model dynamically or by reducing the packet size adaptively. Without regard to adaptive handling, the application requirement remains the same. Adaptive traffic handing consists of two major functions, multiple sets for (*m*,*k*)-firm stream and dynamic payload adjustment.

First, $S_{i}$ is redefined as {$node_{i}$, $m_{i}$, $k_{i}$, $D_{i}$, $DBP_{i}$} where ($m_{i}$, $k_{i}$) ∈ {$m_{i}^{1}$, $k_{i}^{1}$), …($m_{i}^{s}$, $k_{i}^{s}$} as shown in Algorithm 2. By redefining the (*m*,*k*)-firm stream as sets, a (*m*,*k*)-firm stream is composed with loose (*m*,*k*)-firm which conforming the initial requirement. Because this procedure is performed whenever no more path is available in adaptive routing algorithm, it aims to take loose (*m*,*k*)-firm requirement. In $S_{i}$, the lower index has, the looser (*m*,*k*)-firm requirement set. In order to maintain consistency for requirement, each element in $S_{i}$ should keep the condition of Equations (1) and (2). One of the elements in the set ($m_{i}$, $k_{i}$) is selected by comparing the DBP value at consecutive moments in time, i.e., at *t* and t+1. The selection procedure is dependent on the DBP value.
**Algorithm 2** Adaptive Traffic Handling at node *i*.1: $DBPT_{i}^{t}$← DBP value of node *i* at time *t*2: *e*← Current index in {$m_{i}^{1}$, $k_{i}^{1}$), …($m_{i}^{s}$, $k_{i}^{s}$} ▹ The larger index means the looser (*m*,*k*)-firm requirement3: *k*← Parameter to reduce packet size 4: **if**$DBPT_{i}^{t}$ < 0 **then**5:  **if**
$DBPT_{i}^{t+1}$ < 0 **then**▹ if two consecutive DBP values are negative6:   **if**
*e* != *s*
**then**▹ If any available traffic set for (*m*,*k*) is available7:    ($m_{i}$, $k_{i}$) = ($m_{i+1}$, $k_{i+1}$) ▹ Change traffic set for next loose (*m*,*k*)8:   **else**▹ If any available traffic set for (*m*,*k*) is not available9:    ($m_{i}$, $k_{i}$) = ($m_{s}$, $k_{s}$) ▹ Set the traffic as the least (*m*,*k*) requirement10:    payload_size←payload_size × $\frac{1}{k}$▹ Traffic shaping by reducing packet size11:    Payload_Size_Changed == TURE ▹ Set the flag12:   **end if**13:  **else**▹ if only one DBP values are negative14:   ($m_{i}$, $k_{i}$) = ($m_{i-1}$, $k_{i-1}$) ▹ Set the traffic as the next strict (*m*,*k*) requirement15:  **end if**16: **else**▹ If the second DBP value become postive17:  **if**
$DBPT_{i}^{t+1}$ > 0 **then**18:   **if**
*e* == *s* && Packet_Size_Changed == TURE **then**▹ If the current set index is the largest  value19:    payload_size←payload_size × *k*▹ Restore the packet size20:   **end if**21:  **else**22:   ($m_{i}$, $k_{i}$) = ($m_{i-1}$, $k_{i-1}$) ▹ Set the traffic as the next strict (*m*,*k*) requirement23:  **end if**24: **end if**

- If the two values are negative as shown in lines 3 and 4, the current requirements of ($m_{i}$, $k_{i}$) are relaxed to ($m_{i+1}$, $k_{i+1}$), where the current index does not reach the maximum value. This is to allow more missed packets in $S_{i}$ by loosing (*m*,*k*)-firm requirement. This kind of adjustment is available when any subset is available. Otherwise, if the current index is the maximum, another approach to reduce the payload size is applied.
- If the second DBP value becomes positive, the index for ($m_{i}$, $k_{i}$) increases to make current (*m*,*k*)-firm requirement strict because packet delay is not passed beyond deadline. This is, recovery procedure is invoked according to current positive DBP value.
- If the two consecutive DBP values are positive, two different tasks are performed according to the current index according to current index value. If the current index reaches the maximum value and the packet size is changed, the previous payload size is restored. Otherwise, ($m_{i}$, $k_{i}$) is set to close to ($m_{1}$, $k_{1}$) by decreasing the index number.
- If the DBP value becomes positive every time, the algorithm for adaptive traffic handling is not executed because the ($m_{i}$, $k_{i}$)-firm requirement is met by adaptive QoS routing.

**Figure 2:**
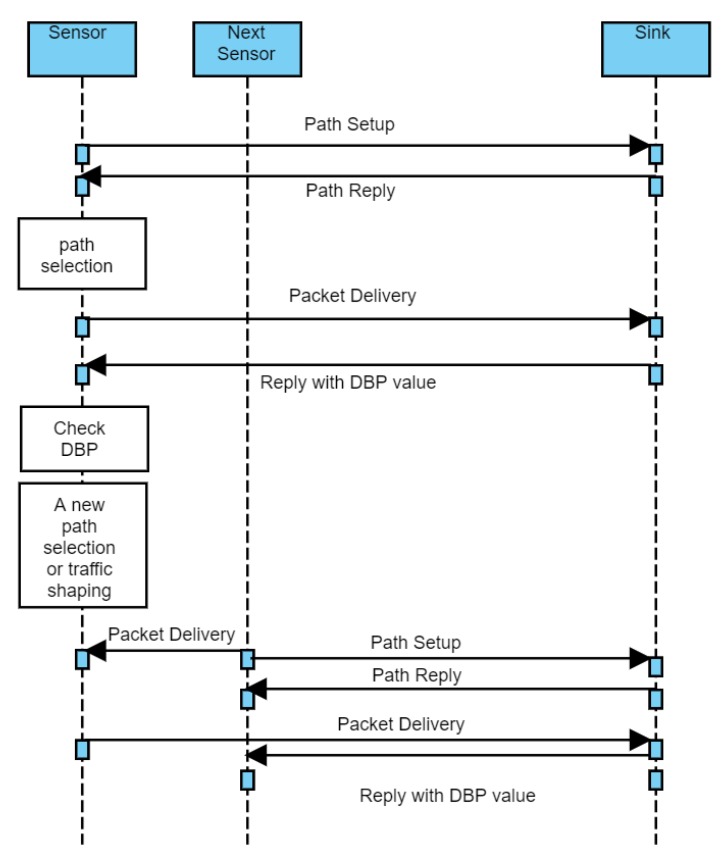
Overview of the proposed real-time model.

As for all algorithms, Figure 2 shows the time sequence diagram for the proposed scheme. At first, path setup procedure is initiated. After receiving reply from the sink node, a sensor node starts delivering packets along the selected path according to (*m*,*k*)-firm requirements. The timeliness of packet is measured in a sink node and reported to a sensor node. According to this DBP value, a node decides whether new path is demanded or traffic shaping is required. If a next node detects event, a packet is delivered to the previous node and new path is searched. A previous node delivers packet from the next node along the path between itself and sink node. After new path is established, packet is delivered from current node to sink.

## 4. Performance Evaluation

This section presents the results of the simulation of the proposed scheme. We used the latest real-time routing protocol known as MK-AG [33] with the OPNET Modeler to simulate and analyze the proposed scheme. MK-AG selects a path based on multiple criteria, which are combined by the Analytical Hierarchical Process (AHP) in conjunction with the Gray Relational Analysis (GRA). However, MK-AG considers the DBP value of the stream without regard to the requirements of the particular application.

Table 1 provides details of the values we used in our simulation. The nodes are placed in a terrain sized 1000 × 1000 m. One hundred nodes were used, of which 10 nodes were placed in the form of a grid, and the remaining 90 were randomly placed. Thus, the node density is determined arbitrarily. The results took the simulation environment into consideration. The transmission and receiving power consumption of a sensor node is 24.92 and 19.72 mJ, respectively, per byte. In order to generate traffic, we employ video traffic model in OPNET. A source node is selected randomly. A source node generate video traffic during random period. After period is over, next node in the source’s transmission range is chosen. If the selected node cannot find the next source node, node is selected in backward direction continuously.

The sink is located in the lower middle region of the field such that the end-to-end hop-count ranges from 4 to 9 hops with an average of 6 hops. The deadline for a real-time packet on each node was set to the average-link-delay × the number of shortest hops, respectively. We conduct the separate simulation to obtain average-link-delay. The evaluation result is presented as the stream dynamic success ratio (SDSR) based on stream dynamic failure ratio (SDFR) of the application. SDFR is the ratio sum of dynamic failures to total group of packets as much as *k* in the stream (total_number_of_packets/*k*). A dynamic failures happens if fewer than *m* out of any *k* consecutive packets are not delivered within their deadlines. We compute SDSR in following equation

(3) $$\begin{array}{c} SDSR=(1-SDFR)\\ where\\ SDFR=\frac{sum\_of\_dynamic\_failures}{number\_of\_groups} \end{array}$$

Additionally, we express the efficiency of the proposed scheme in terms of the network lifetime if the lifetime is prolonged.

For the (*m*,*k*)-firm stream for each $S_{i}$, the number of available stream sets, *s*, is set to 3. In addition, the value of *t*, which is used to monitor the DBP values, is set to 3. The parameter for reducing the packet size, *k* is set to 2 in Algorithm 2 such that a payload of 64 bytes is reduced to 32. As the background traffic, we employed three Constant Bit Rate (CBR) traffic streams and varied the amount of traffic to ensure the network is congested. Moreover, the source and destination of the background traffic was randomly chosen and was continuously changed after the predetermined duration. We calculate 95% confidence interval of average SDSR for 20 times evaluations with different 206 seed numbers for source and destination selection. Each simulation is conducted during 1000 s.

### Simulation Results

**Figure 3:**
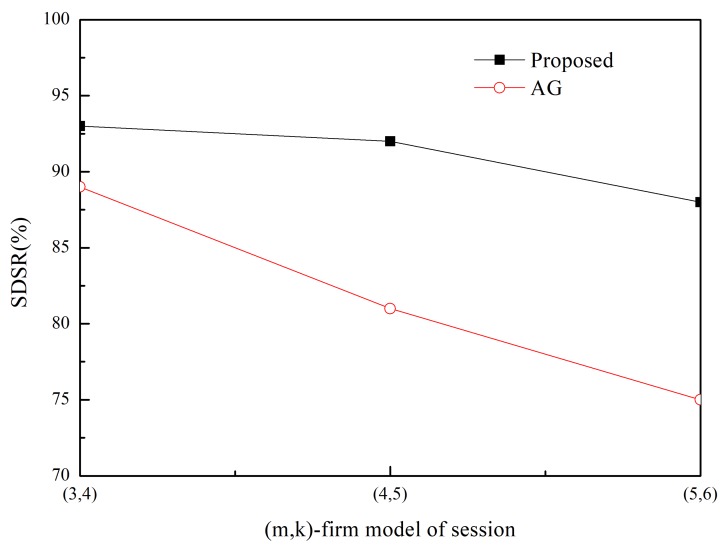
SDSR for (2,3)-firm application.

**Figure 4:**
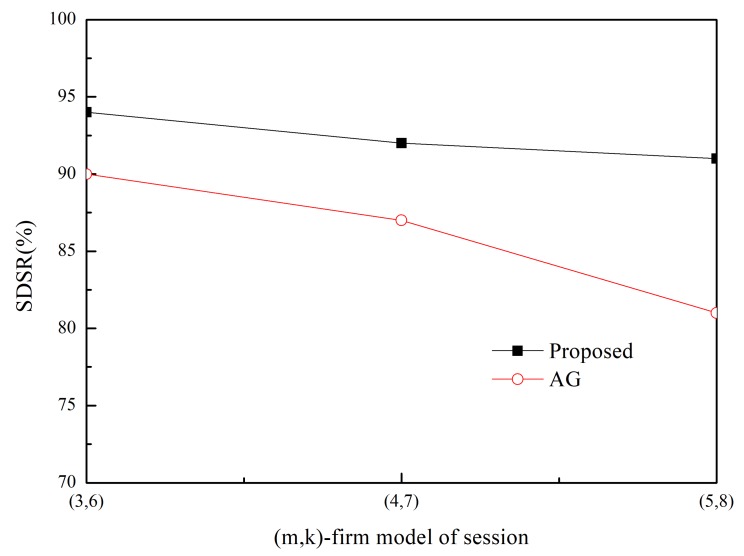
SDSR for (2,5)-firm application.

The SDSR of the proposed scheme and that of the MK-AG routing protocol (denoted AG) are compared in Figure 3 and Figure 4. Two respective (*m*,*k*)-firm streams were considered for application. Figure 3 shows the results of (2,3) as a more strict requirement than (2,5) in Figure 4. For each application, we compare three different (*m*,*k*)-firm streams. In other words, for each (*m*,*k*)-firm stream, three respective streams are composed. For example, the (3,4)-firm session stream consists of the set of {(3,4),(6,8), (9,12)}. Following the same approach, all sessions of the (*m*,*k*)-firm streams are defined as the set of three (*m*,*k*)-firm streams according to Equations (1) and (2).

Figure 3 and Figure 4 show that the SDSR of the strict requirement is lower than that of the less strict requirement. The same pattern is observed for the session requirements. That is, the highest SDSR is obtained for (3,6) session traffic in the (2,5)-firm application. In addition, the SDSR of the proposed scheme is higher than that of the MK-AG routing protocol. In Figure 3, the difference between the two schemes is larger where a strict requirement is demanded. The main reason for this gap is the adaptive routing protocol. Even though MK-AG takes into account the speed for real-time delivery, the delivery ratio on the link, and the remaining energy, it occasionally happens that a different path is not selected according to the DBP value. This implies that a different path is not selected when the multi-criteria approach is followed. In addition, the selection of a different path by the static comparison matrix in the AHP is time consuming. On the other hand, the proposed scheme makes use of multiple path selection according to the DBP value. Thus, because path selection is accomplished whenever the DBP value becomes negative, higher SDSR is achieved. The other important factor that affects the performance of the application is to take $DBP_{ObjID}$ in $DBP_{ObjID}$. Because MK-AG only focuses on the session requirement, its compliance with the application requirements is less successful. However, the proposed scheme overcomes this problem by assigning a higher priority to $DBP_{ObjID}$ in $DBP_{ObjID}$ than $DBP_{i}$ in $S_{i}$ in Algorithm 1; hence, it promptly meets the requirement of the application according to $DBP_{ObjID}$.

In addition to using two schemes, adaptive traffic handling is performed according to the strict requirement. By using these diverse scenarios, we found that adaptive traffic handling was not invoked in too many cases, as would be expected for the strict (*m*,*k*)-firm requirement. More specifically, the adaptive traffic handling algorithm was executed 25 times in the (5,6)-firm session in the (2,3)-firm application, whereas this was not observed to occur in the (3,6)-firm session in the (2,5)-firm application. Among the 25 executions, five requests to change the packet size were detected. On the other hand, because the MK-AG routing protocol does not include an algorithm to adjust the traffic, more packets would be able to miss the deadline even though the shortest path is established. The effect of the traffic-handling algorithm is highlighted when strict requirements are used.

Usually control overhead may affect SDSR because high volume of control messages make network delay long. In the proposed scheme, control message includes RREQ and RREP during Path_Search_Probing(i,sink) procedure in Algorithm 1. Furthermore, a sink node periodically sends control message to a source node with $DBP_{ObjID}$ and $DBP_{i}$. The former has negative effect in SDSR because it is based on the broadcast. However, its impact to SDSR is not great because the path search procedure is performed at the initial stage. Furthermore, control message between sink and source node is transmitted in a unicast way. Thus, its impact to SDSR is also limited. Similarly, control overhead of MK-AG is limited to path search and periodical reporting. In addition, there is no difference on the control overhead between streams because control message in the proposed scheme consists of path search and reporting. This implies that the performance gap between two protocols is observed by effectiveness of the adaptive path selection and traffic shaping. Specially, traffic shaping plays a great role in strict requirement by providing several alternative path and (*m*,*k*)-firm set.

**Figure 5:**
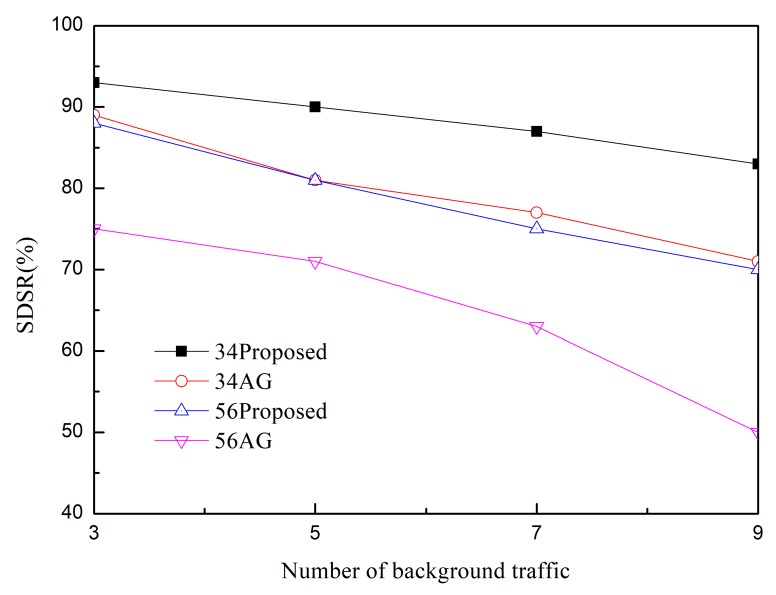
SDSR in (2,3)-firm application.

**Figure 6:**
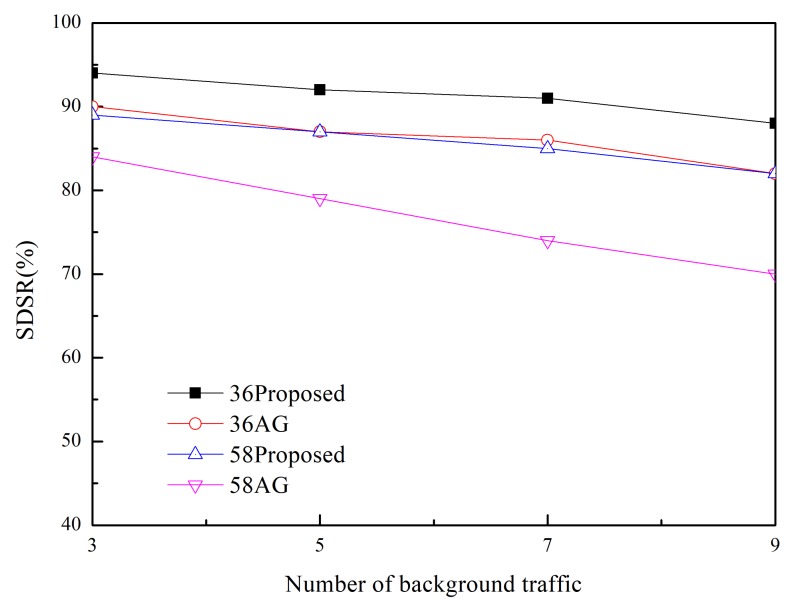
SDSR in (2,5)-firm application.

We evaluated the performance when the network is congested by measuring the SDSR of the application as a function of the amount of background traffic. The results are plotted in Figure 5 and Figure 6 for different (*m*,*k*)-firm sessions and applied schemes. For instance, 56AG represents (5,6)-firm stream session supported by MK-AG. The results of this evaluation exhibited a similar pattern as a function of different requirements. In all cases, the SDSR decreases as the amount of background traffic increases. The increased background traffic caused the network to become congested; therefore, more packets missed the deadline or were discarded. As compared to the MK-AG protocol, the proposed scheme is robust against congestion; consequently, adaptive path selection and traffic adjustment contribute to finding alternative paths. On the other hand, even though MK-AG takes congestion into account by considering the link delivery ratio, it does not compensate for lost packets as a result of congestion. Moreover, the static comparison matrix does not allow the source to change the path accordingly. As compared to light background traffic condition in Figure 3 and Figure 4, the high impact of control overhead is observed in Figure 5 and Figure 6. This is caused by the delay of control message at intermediate node as well as packet loss. These two features can lead to prevent establishing multiple paths and deliver the DBP values in early time. As a result, path selection and traffic shaping can be perform later than previous case, that is, light traffic condition. Thus, more packets missed deadline so lower SDSR is measured. This negative effect is observed in strict (*m*,*k*)-firm requirement more frequently.

**Figure 7:**
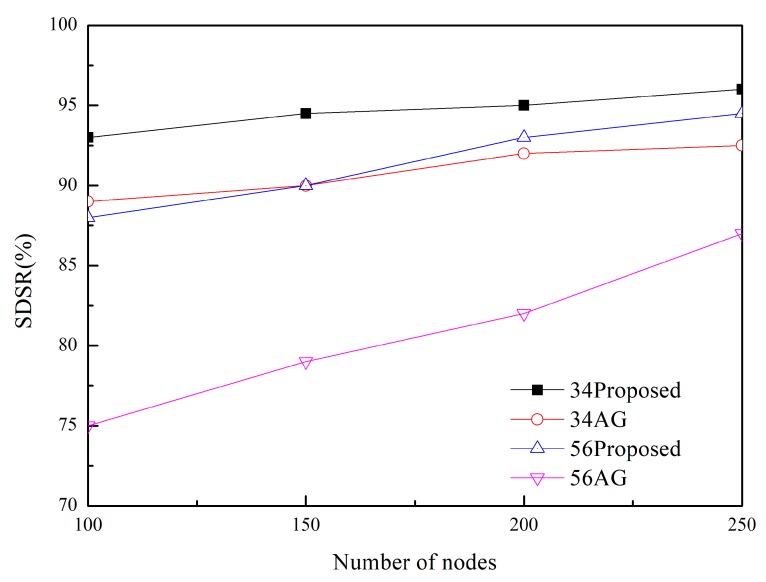
SDSR in (2,3)-firm application as a function of node density.

**Figure 8:**
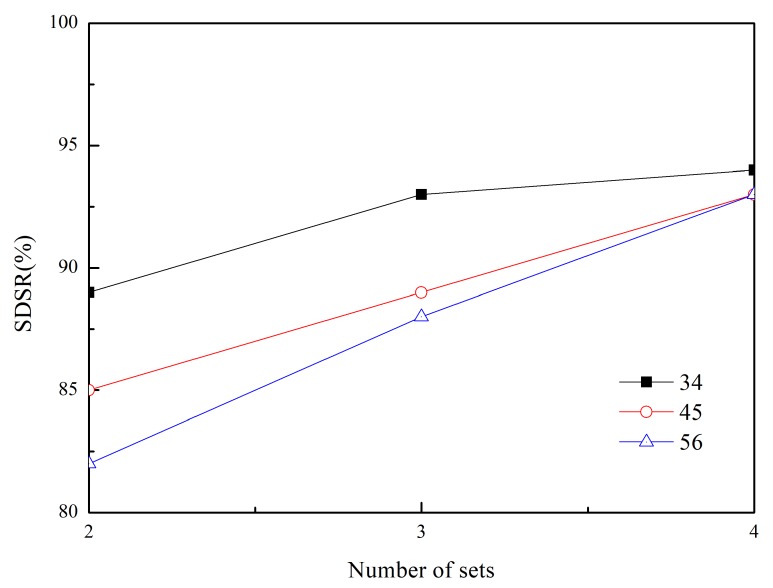
SDSR in (2,3)-firm application as a function of (*m*,*k*)-firm sets.

In addition to conducting a comparative study with the existing scheme, we conducted the simulation by varying the network environment and parameters in the proposed scheme. Figure 7 shows the SDSR of the (2,3)-firm application as a function of the number of nodes. These results show that an increase in the number of nodes contributes to improving the SDSR. Because the node density is related to the number of paths, a large number of nodes enable more paths to be established between nodes. This means that the options of load balancing to prevent congestion and increased opportunities to change the path become available. Based on this analysis, great improvement is observed when using a more strict requirement such as a (5,6)-firm stream. The last simulation result in Figure 8 shows the impact of the number of (*m*,*k*)-firm sets on each session. We measured the SDSR by varying the number of sets from 2 to 4 in the adaptive traffic handling procedure in Algorithm 2. Higher SDSR is observed for a larger number of sets whereas the lowest values are measured for two sets. Because the larger number of levels provides a greater possibility to maintain a positive DBP value, enhanced SDSR values are obtained in all cases. In particular, a stricter (*m*,*k*)-firm streams benefits from a large number of sets.

**Figure 9:**
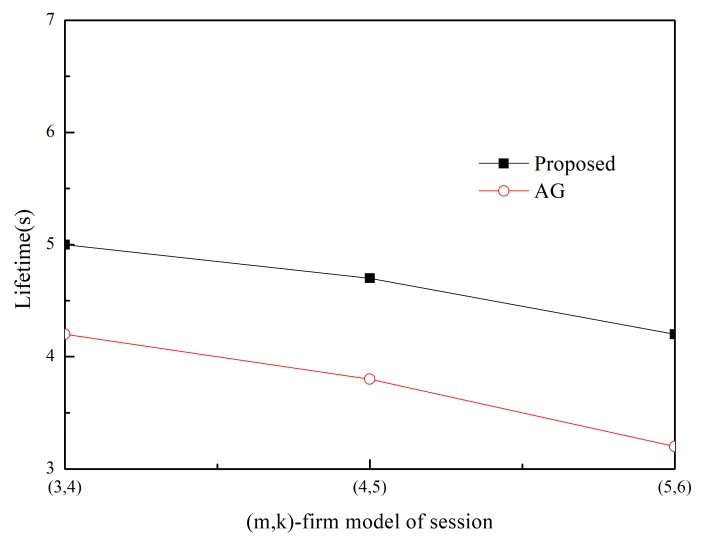
Lifetime in (2,3)-firm application as a function of (*m*,*k*)-firm sets.

**Figure 10:**
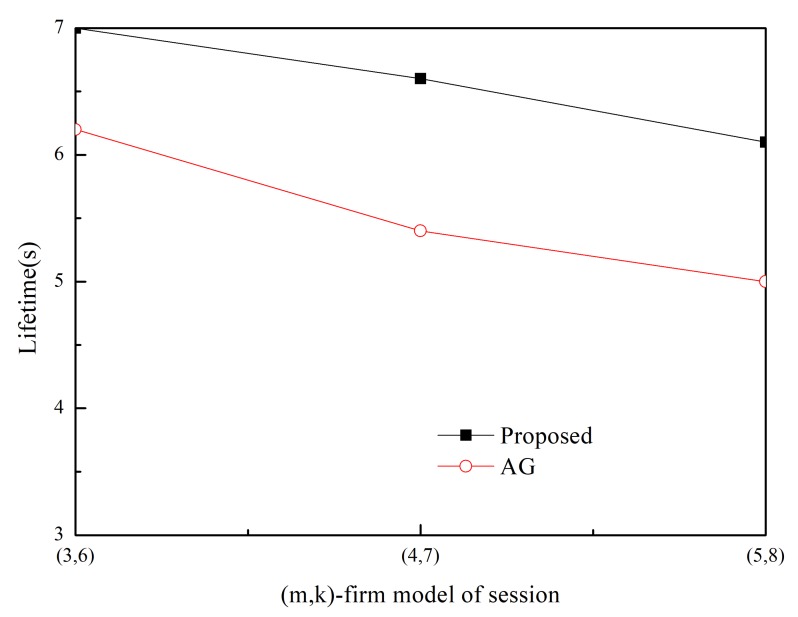
Lifetime in (2,5)-firm application as a function of (*m*,*k*)-firm sets.

We measure the network lifetime of the proposed and existing scheme in two separate (*m*,*k*)-firm applications in Figure 9 and Figure 10. In this paper, network lifetime is defined as elapsed time until the first sensor node or group of sensor nodes in the network runs out of energy. Because a node’s energy consumption in the routing protocol is mostly dependent on the how many paths are established and how many packets are transmitted on a node, longer network lifetime than MK-AG is observed in the proposed scheme which is based on multiple paths which lead to distribute the packets along the multiple paths according to DBP values. In addition, adaptive path selection algorithm leads to prevent significant battery drain on nodes along the path. In the aspects of (*m*,*k*)-firm requirement, strict requirement is usually met by one or at most two paths. This limited number of path accelerates the battery drain on nodes. Therefore, short network lifetime is naturally observed in more strict requirement. In the other hand, network lifetime can be affected by the number of control message. Because our approach is based on path search through flooding, larger number of control messages lead to reduce network lifetime. In addition, reply message with DBP value is proportional to number of sent message. Despite of this shortcoming, longer network lifetime is achieved by the adaptive routing and traffic shaping. This implies that network lifetime is more dependent on the data packet than control one.

**Figure 11:**
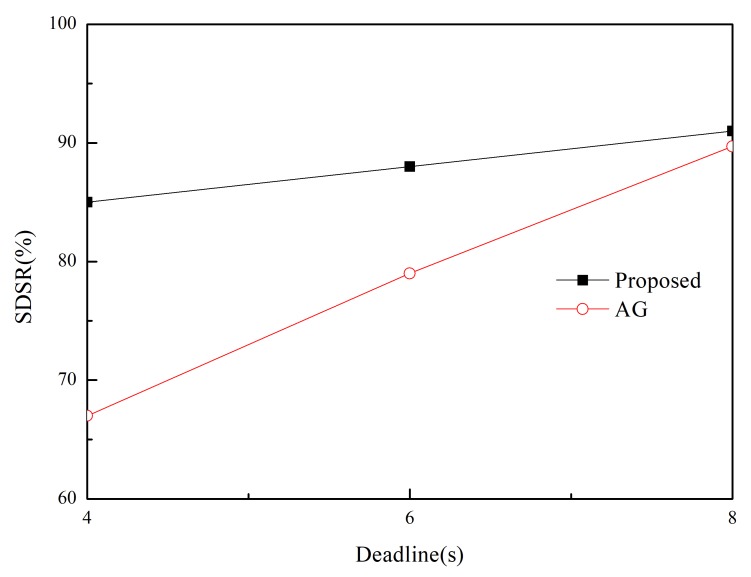
SDSR in (2,3)-firm application as a function of deadline.

**Figure 12:**
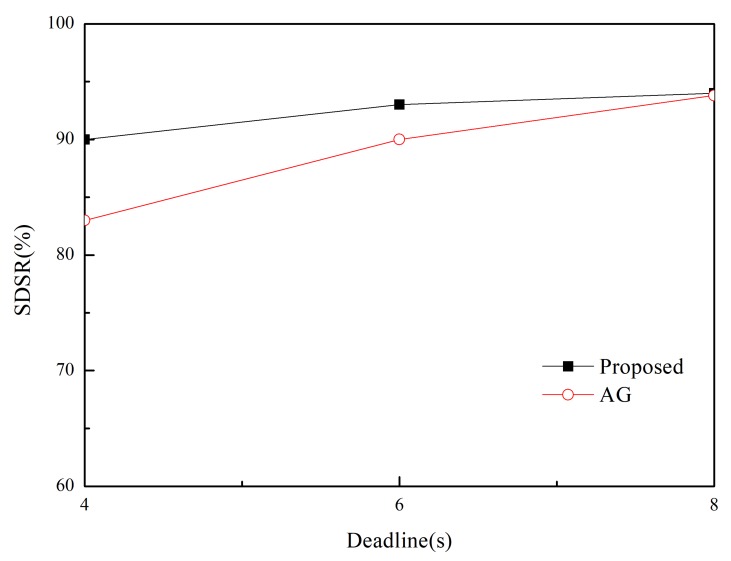
SDSR in (2,5)-firm application as a function of deadline.

Finally, we conduct the simulation for SDSR for varying deadline as illustrated in Figure 11 and Figure 12. In both figures, lower SDSR is observed shorter deadline is assumed in both schemes. However, higher SDSR is measured in the proposed scheme rather than MK-AG. Due to adaptive path selection and traffic adjustment algorithm lead to find the more suitable path than MK-AG. Even though larger gap between two schemes are measured in short deadline, almost identical SDSR is observed in the long deadline. This implies that two schemes are able to meet real-time delivery in appropriate way. In the aspects of (*m*,*k*)-firm requirement, less strict (*m*,*k*)-firm requirement induces higher SDSR than strict one. If the requirement is not strict, more available paths are likely to be established. This is mainly because packet loss and queuing delay caused by congestion are reduced.

As a result, we can summary the performance of the proposed scheme and MK-AG protocol in Table 2. We present the relative value according to (*m*,*k*)-firm requirement and given the metrics. As described in the Table 2, our proposed scheme is observed to provide more comparative performance than the existing scheme.

## 5. Conclusions

In this paper, we presented a new real-time routing protocol and traffic handling algorithm for (*m*,*k*)-firm streams in wireless multimedia sensor networks. The application and session traffic were modeled by (*m*,*k*)-firm requirements and monitored by specifying a deadline for packet transmission. The current status of the (*m*,*k*)-firm requirement in both applications and sessions was used to choose an alternative path and adjust the traffic. Simulation results were provided to prove the efficiency of the proposed scheme compared to an existing scheme. The impact of the parameters was also analyzed.

Related to this scheme, we need to exploit real-time routing protocol to improve the energy efficiency of the proposed scheme. Furthermore, multimedia coding and compression scheme for (*m*,*k*)-firm streams will be studied. In addition, the improved adaptive path selection algorithm will be studied to reflect the latest end-to-end delay as well as predict next event. Finally, it will be required to introduce practical potential application such as object tracking to utilize the proposed mechanism.

## Figures and Tables

**Table 1:** Simulation environment setting.

Parameter	Value(s)
Terrain	(1000 m, 1000 m)
Number of nodes	100 Nodes
Node placement	Uniform & Random placement
Transmission range (m)	40 m
PHY and MAC protocol	802.15.4 PHY & MAC
Bandwidth	250 Kb/s
Payload size	64 bytes
Reduced payload size	32 bytes
Energy consumption (Tx)	24.92 mJ per 1 byte
Energy consumption (Rx)	19.72 mJ per 1 byte
Traffic model	Video traffic
Frame Interval Time	10 frame/s

**Table 2:** Summary for SDSR.

Parameters	(*m*,*k*)-Firm Type	Proposed	MK-AG
High Node Density	Strict	High	High
	Loose	High	Medium
Low Node Density	Strict	High	High
	Loose	High	Low
Network Lifetime	Strict	Medium	Low
	Loose	High	Medium
Short Deadline	Strict	Medium	Low
	Loose	High	Medium
Long Deadline	Strict	High	High
	Loose	High	High
